# Long Noncoding RNA *UCA1* in Gastrointestinal Cancers: Molecular Regulatory Roles and Patterns, Mechanisms, and Interactions

**DOI:** 10.1155/2021/5519720

**Published:** 2021-04-10

**Authors:** Suaidah Ramli, Maw Shin Sim, Rhanye M. Guad, Subash C. B Gopinath, Vetriselvan Subramaniyan, Shivkanya Fuloria, Neeraj K. Fuloria, Ker Woon Choy, Sohel Rana, Yuan Seng Wu

**Affiliations:** ^1^Department of Pharmaceutical Life Sciences, Faculty of Pharmacy, University of Malaya, Kuala Lumpur 50603, Malaysia; ^2^Department of Biomedical Science and Therapeutics, Faculty of Medicine and Health Science, Universiti Malaysia Sabah, Kota Kinabalu 88400, Sabah, Malaysia; ^3^School of Bioprocess Engineering, Universiti Malaysia Perlis, Arau 02600, Perlis, Malaysia; ^4^Institute of Nano Electronic Engineering, Universiti Malaysia Perlis, Kangar 01000, Perlis, Malaysia; ^5^Department of Pharmacology, School of Medicine, Faculty of Medicine, Bioscience and Nursing, MAHSA University, Jenjarom, Selangor 42610, Malaysia; ^6^Faculty of Pharmacy, AIMST University, Bedong, Kedah 08100, Malaysia; ^7^Department of Anatomy, Faculty of Medicine, Universiti Teknologi MARA, Shah Alam, Sungai Buloh 47000, Selangor, Malaysia; ^8^Department of Pharmacy, Faculty of Biological Science and Technology, Jashore University of Science and Technology, Jashore-7400, Bangladesh; ^9^Department of Biochemistry, School of Medicine, Faculty of Medicine, Bioscience and Nursing, MAHSA University, Jenjarom, Selangor 42610, Malaysia

## Abstract

The rising trend of gastrointestinal (GI) cancer has become a global burden due to its aggressive nature and poor prognosis. Long noncoding RNAs (lncRNAs) have recently been reported to be overexpressed in different GI cancers and may contribute to cancer progression and chemoresistance. They are featured with more than 200 nucleotides, commonly polyadenylated, and lacking an open reading frame. LncRNAs, particularly urothelial carcinoma-associated 1 (*UCA1*), are oncogenes involved in regulating cancer progression, such as cell proliferation, invasion, migration, and chemoresistance, particularly in GI cancer. This review was aimed to present an updated focus on the molecular regulatory roles and patterns of lncRNA *UCA1* in progression and chemoresistance of different GI cancers, as well as deciphering the underlying mechanisms and its interactions with key molecules involved, together with a brief presentation on its diagnostic and prognostic values. The regulatory roles of lncRNA *UCA1* are implicated in esophageal cancer, gastric cancer, pancreatic cancer, hepatobiliary cancer, and colorectal cancer, where they shared similar molecular mechanisms in regulating cancer phenotypes and chemoresistance. Comparatively, gastric cancer is the most intensively studied type in GI cancer. LncRNA *UCA1* is implicated in biological roles of different GI cancers via interactions with various molecules, particularly microRNAs, and signaling pathways. In conclusion, lncRNA *UCA1* is a potential molecular target for GI cancer, which may lead to the development of a novel chemotherapeutic agent. Hence, it also acts as a potential diagnostic and prognostic marker for GI cancer patients.

## 1. Introduction

Gastrointestinal (GI) cancer has become one of the major challenges in the health sector in recent decades. GI cancer is a group of cancers that affect the GI tract, such as esophagus, stomach, gallbladder, liver, biliary tract, small intestine, and large intestine [1, 2]. In 2018, GI cancer contributed 26% among all cancer cases and 35% of cancer-causing death worldwide [3]. There are five major GI cancers, namely, gastric cancer (GC), hepatobiliary cancer, esophageal cancer (EC), pancreatic cancer (PC), and colorectal cancer (CRC), accounting for approximately 1 million, 840,000, 570,000, 460,000, and 1.7 million new cases were reported in 2018, respectively [4]. Comparatively, EC, GC, and liver cancer (LC) are predominant in Asian population, whereas CRC shows more incidence in Europe and North America [3]. Apart from that, GI cancer shows a reduced 5-year survival rate and a poor prognosis at the late stage of cancer [5]. Generally, several factors have been reported to be the contributing risk factors for GI cancer, including tobacco smoking, alcohol consumption, diet, and obesity and infections, such as *Helicobacter pylori* in GC and hepatitis virus in LC [3, 6, 7].

With the recent advancement in RNA sequencing technology transcriptome knowledge, there are increased interests in long noncoding RNAs (lncRNAs) as they play an important role in tumorigenesis, particularly gene regulation [8, 9]. LncRNA is characterized by possessing more than 200 nucleotides that would not be translated into protein [10]. It can be found in both nucleus and cytoplasm where the chromatin remodeling, transcriptional regulation, and RNA processing take place in the nucleus, while its interaction with mRNA and signaling pathway occurs in the cytoplasm [11, 12]. One of the reported cancer-related lncRNAs is urothelial carcinoma-associated 1 (*UCA1*) that was first discovered in 2006 as it was found to be overexpressed in bladder cancer (BC) cells, a cancer close to but not belonged to GI cancer [13]. It belongs to human endogenous retrovirus H family and is located at 19p13.12 of the chromosomes positive-strand with three exons and two introns [13]. To date, three lncRNA *UCA1* isoforms produced by RNA splicing have been discovered, and each of them with different sizes, including 1.4, 2.2, and 2.7 kb [14, 15]. Among them, 1.4 kb lncRNA *UCA1* is the most assessed and abundant isoform, while 2.2 kb isoform is relatively more participated in chemoresistance [14]. For instance, Wang et al. showed that lncRNA *UCA1* significantly associated with cancer chemoresistance toward cisplatin, gemcitabine, 5-fluorouracil, tamoxifen, and imatinib. Interestingly, the chemosensitivity of these drugs was significantly increased when lncRNA *UCA1* was silenced [16].

Apart from these, lncRNA *UCA1* has been detected to be overexpressed in various cancers, particularly GI cancers, such as CRC, esophageal squamous cell carcinoma (ESCC), hepatocellular carcinoma (HCC), and GC [17–19]. Among lncRNAs, lncRNA *UCA1* has been demonstrated to have significant regulatory roles in cancer progression, including cell proliferation, invasion, migration and metastasis, and chemoresistance in BLS-211 BC cells [13]. In the last decade, the regulatory roles of lncRNAs have been intensively investigated in which most studies have suggested that the mechanistic pathways underlying the regulatory roles of lncRNA *UCA1*. In this context, its interaction with the key genes or proteins is the key causative factor that leads to the development of GI cancer.

Therefore, this review aims to provide a detailed insight into the regulatory roles of lncRNA *UCA1* in GI cancer progression and chemoresistance, as evidenced in preclinical and clinical studies. In addition, it also discusses various molecular mechanisms underlie and the key molecules involved, intending to present its potential as a novel molecular target, as well as a diagnostic and prognostic marker for GI cancer.

## 2. LncRNA *UCA1*

**Figure 1:**
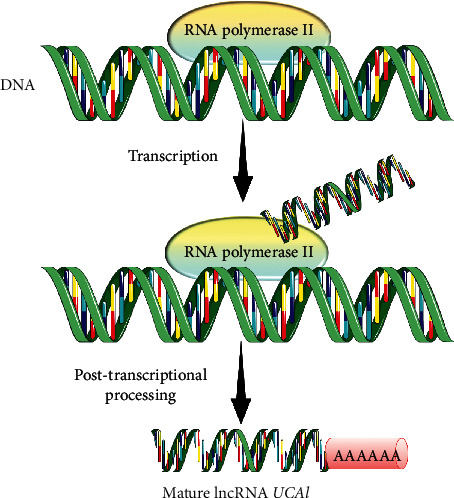
Biogenesis of lncRNA *UCA1*. LncRNA *UCA1* is produced by transcriptional process mediated by RNA polymerase II from DNA template. It then undergoes special posttranscriptional processing events, including 5′-capping, splicing, polyadenylation, and chemical base modification, to become a mature form.

Over the past few years, there is a bloom of transcriptome studies associated with the advancement in RNA sequencing technology, which enables the view of the complexity of eukaryotic gene expression [20]. This advanced technology leads to the discovery of lncRNAs [21]. More than 98% of the genomes transcribed into ncRNAs are categorized, either as structural RNAs or regulatory RNAs, where lncRNA is classified under regulatory RNAs [22]. LncRNAs are discovered as an important new player in cell differentiation and development, as well as organogenesis and genomic imprinting [23, 24]. Additionally, most lncRNAs, including lncRNA *UCA1*, are much like mRNAs where they are transcribed by RNA polymerase II with similar chromatin states to mRNAs, and they usually 5′capped, spliced, and polyadenylated [25, 26]. The biogenesis of lncRNA *UCA1* is illustrated in Figure 1.

It has been reported that several lncRNAs participate in the special processing events, including DNA organization. In this event, genomic DNA is packed in the nucleus with a special genomic organization, depending on both histone and chromatin modifications that are regulated by epigenetic complexes and affect the transcriptional activity [27, 28]. For instance, lncRNA metastasis-associated lung adenocarcinoma transcript 1 (*MALAT1*) and lncRNA nuclear enriched abundant transcript 1 (*NEAT1*) are localized at the nuclear speckles and nuclear paraspeckles, respectively, after processing at 3′ ends by RNA polymerase II to form tRNA-like small RNA products and mature lncRNAs [25, 29, 30]. However, the exact DNA organization for lncRNA *UCA1* remains to be confirmed. Functionally, lncRNAs are involved in chromatin and epigenetic modifications [31, 32]. LncRNA *UCA1* also acts as an miRNA decoy and miRNA sponge, which sequester miRNA intracellularly and compete with other genes for miRNA binding, leading to an increased level of miRNA target gene expression [1, 33].

Furthermore, lncRNA has also shown to play an important role in embryogenesis where it has been identified to be upregulated after 28 weeks of gestational in the tissue of heart, urinary bladder, and uterus, but downregulation is detected in liver, kidney, lung, spleen, intestine, stomach, skin, and cervix. In adult tissues, lncRNA *UCA1* expression is relatively conserved at a low expression level, except for heart, spleen, and placenta [34]. In short, the ideal expression of lncRNA *UCA1* is remarkably essential for cell growth and development, particularly in embryogenesis stage.

## 3. Molecular Regulatory Roles, Patterns, Mechanisms, and Interactions of LncRNA *UCA1* in Different Gastrointestinal Cancers

**Figure 2:**
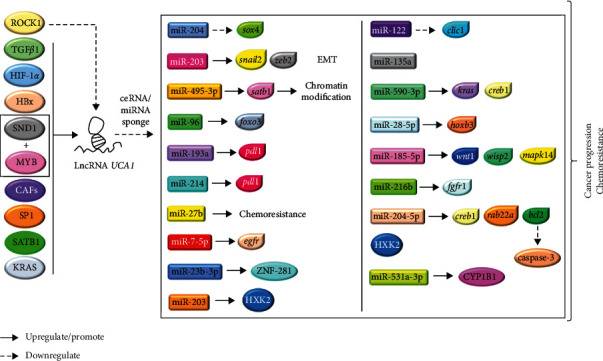
Overview of the upstream and downstream regulation of lncRNA *UCA1* on miRNAs, genes, and proteins in GI cancer. LncRNA *UCA1* could be induced by TGF-*β*1, HIF-1*α*, HBx, SND1-MYB complexes, CAFs, SP1, SATB1, and KRAS proteins, while ROCK1 can repress its expression. In turn, it acts as a ceRNA and an mRNA sponge that can reduce miRNA expression, which further downregulate its mediating gene expression. Collectively, lncRNA *UCA1* regulates this interaction network to promote cell proliferation, metastasis, and chemoresistance in different GI cancers.

**Figure 3:**
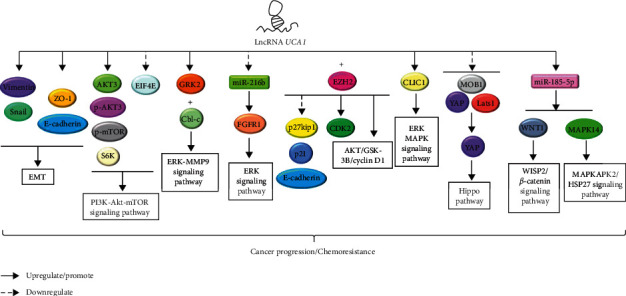
The signaling pathway associated with lncRNA *UCA1* in GI cancer. LncRNA *UCA1* induces EMT by regulating EMT key proteins. It also promotes the activation of PI3K/Akt/mTOR signaling pathway, ERK/MMP9 signaling pathway, ERK signaling pathway, Hippo pathway, WISP2/*β*-catenin signaling pathway, and MAPKAPK2/HSP27 signaling pathway by regulating their key proteins. Additionally, lncRNA *UCA1* also interacts with EZH2 to regulate protein expression.

It has been reported that high expression levels of lncRNA *UCA1* are detected in GI cancer cells [35, 36]. Thus, lncRNAs may play an important role for GI tumorigenesis. The positive association of lncRNA *UCA1* with the overall survival of GI cancer patients was revealed in a meta-analysis [35]. The pooled result of 14 studies indicated that poor overall survival in patients with digestive malignancies was associated with lncRNA *UCA1* overexpression [35]. Since then, different studies were conducted to further discover the association between GI cancer and lncRNA *UCA1* as well as identify the possible mechanisms responsible for GI cancer progression. In this review, the expression pattern, regulatory roles and patterns, mechanistic pathways, and interactions of key molecules that are associated with lncRNA *UCA1* in GI cancer progression and chemoresistance, including EC, GC, hepatobiliary cancer, PC, and CRC, are summarized (Table 1). A brief insight of the potential role of lncRNA *UCA1* as a diagnostic and prognostic marker, wherever applicable in different GI cancers, is also presented. The interaction of lncRNA *UCA1* that affects the target gene expression of miRNAs and activation of pivotal signaling pathway are illustrated in Figures 2 and 3, respectively.

### 3.1. Esophageal Cancer

In ESCC patients, the most predominant deadly types of EC, lncRNA *UCA1* has been reported to be overexpressed and contributed to poor prognosis [37]. Jiao et al. showed that lncRNA *UCA1* was strongly associated with EC cell proliferation by functioning as a competing endogenous RNA (ceRNA) to regulate the expression of Sry-related high-mobility group box 4 (Sox4), a target protein of lncRNA *UCA1* [38]. Additionally, lncRNA *UCA1* also can directly interact with miR-204 to reduce miR-204-mediated Sox4 degradation; thus, Sox4 can exert its biological role as a tumor-promoting protein to stimulate EC progression [38]. Apart from that, overexpressed lncRNA *UCA1* could also promote cell proliferation and metastasis by enhancing aerobic glycolysis through Warburg effect [39]. These happened when lncRNA *UCA1* sequestered miR-203, which then increased the levels of hexokinase 2 (HXK2) [39].

Despite several studies have reported a positive correlation between overexpressed lncRNA *UCA1* and tumor progression; however, contradictory findings were reported. For instance, Wang et al. discovered that overexpression of lncRNA *UCA1* suppressed ESCC cell growth via the inhibition of Wnt signaling pathway by suppressing *β*-catenin activity [40]. They claimed that lncRNA *UCA1* could reduce the expression of active *β*-catenin protein expression in the cell nucleus and myelocytomatosis proto-oncogene (C-myc), which is a target protein of Wnt signaling pathway in regulating cell cycle. This action ultimately reduced cancer cell proliferation, migration, and invasion [40]. Similarly, Zhu et al. also demonstrated that lncRNA *UCA1* was lowly expressed in EC tissues and plasma exosomes, which is a lipid-bilayer extracellular vesicle used as a cargo system for various molecules, including lncRNAs, for implicating in the pathogenesis of many diseases, including cancer, by regulating intercellular communication. They specifically found that exosomal lncRNA *UCA1* could act as a growth inhibitor in EC as its overexpression inhibited cell proliferation, migration, invasion, and colony formation significantly, as well as tumor growth *in vivo* via direct targeting of high levels of miR-613 [41]. It also acts as a potent diagnostic biomarker for EC, with great sensitivity (86.7%) and specificity (70.2%) [41]. However, these findings need to be further assessed as there is increasing evidence showing that lncRNA *UCA1* acts as an oncogenic lncRNA instead of having tumor-suppressing function. Taken together, further molecular studies of lncRNA *UCA1* should be conducted to elucidate its associated molecular mechanisms of regulatory roles in EC clearly.

### 3.2. Gastric Cancer

GC is one of GI cancers that contribute to high mortality due to late diagnosis [3, 77]. Intriguingly, Gao et al. suggested that lncRNA *UCA1* could be a potential diagnostic and biomarker target in the early stage of GC, owing to the fact that highly expressed lncRNA *UCA1* can be easily found in the plasma of GC patients and therefore provides simplicity for sample extraction [42]. Similarly, it has also been discovered that lncRNA *UCA1* is overexpressed in both GC tumor and cell lines [43]. Moreover, it was also reported to play a role in GC cell migration and invasion via the induction of epithelial-mesenchymal transition (EMT) by competitively binding to miR-203, increasing the expression of its target protein, Zinc Finger E-Box Binding Homeobox 2 (ZEB2) [44].

In addition to miR-203, lncRNA *UCA1* also interacts with miR-495-3p, supporting the role of *UCA1* acting as a ceRNA [45]. Sun et al. reported that lncRNA *UCA1* expression could be regulated by special AT‐rich‐binding protein 1 (SATB1), which was involved in chromatin modification in both MKN-45 and BGC‐823 GC cells [45]. However, lncRNA *UCA1* only regulated the protein levels of SATB1 in MKN-45 GC cells but not in BGC‐823 cells [45]. Thus, further investigation is required to discover the rationale for obtaining such findings.

Similarly, lncRNA *UCA1* has also found to regulate miR-590-3p expression that results in the activation of cAMP-responsive element-binding protein 1 (CREB1), which is an oncogenic protein [46]. In addition, it plays a role in suppressing the immune system of GC cells by elevating the expression of programmed death-1 ligand-1 (PDL1) via sponging miR-193a and miR-214 [47]. In addition, Wang et al. also reported that lncRNA *UCA1* could sponge other miRNAs, for instance, miR-26a and miR-26b, thereby reducing their expression levels [47]. This finding indicated that lncRNA *UCA1* could function as an miRNA sponge to reduce miRNA expression in the cells, subsequently reducing its inhibitory effects on the target protein. On the other hand, reduced ki-67 protein levels and increased levels of cleaved poly [ADP-ribose] polymerase 1 (PARP1) and cleaved caspase 3 were observed in GC cells after lncRNA *UCA1* silencing [47]. However, the exact mechanism of lncRNA *UCA1* in regulating ki-67, PARP1, and caspase 3 is unknown, and further confirmation is required, particularly in identifying miRNAs or proteins associated with the regulation of lncRNA *UCA1*.

In addition, Zuo et al. demonstrated that the induction of high lncRNA *UCA1* expression in GC cells was mediated by transforming growth factor *β*1 (TGF-*β*1) [48]. The overexpressed lncRNA *UCA1* consequently promoted EMT by regulating the expression levels of EMT-related proteins, such as E-cadherin, vimentin, snail, and zonula occludens-1 (ZO-1) [48]. For instance, the mRNA levels of epithelial cell markers, such as E-cadherin and ZO-1, were reduced, while an elevation was observed for mesenchymal cell markers, namely vimentin and snail [48]. This finding indicated that apart from regulating other genes or proteins, lncRNA *UCA1* also can be regulated by other genes or proteins.

Meanwhile, lncRNA *UCA1* has also been reported to regulate phosphatidylinositol-3-kinase (PI3K)/AKT/mammalian target of rapamycin (mTOR) signaling pathway and their downstream mediators [49]. The overexpressed lncRNA *UCA1* increased the expression levels of key molecules in the PI3K/AKT/mTOR signaling pathway, including AKT serine/threonine kinase 3 (AKT3), phosphorylated mammalian target of rapamycin (p-mTOR), and ribosomal protein S6 kinase (S6K), while reducing the eukaryotic translation initiation factor 4E (EIF4E) protein levels in GC cells [49]. Consequently, the regulation of these proteins promoted GC cell growth and proliferation [49]. This finding showed that lncRNA *UCA1* could regulate multiple proteins involved in a signaling pathway.

On the other hand, Wang et al. reported that specificity protein 1 (SP1) promoted the expression of lncRNA *UCA1* in GC cells by binding to the core promoter of *UCA1* [50]. The expressed lncRNA *UCA1* was then activated AKT/GSK-3 B/cyclin D1 axis by interacting with enhancer of zeste homolog 2 (EZH2), a histone methyltransferase [50]. Meanwhile, the interaction of lncRNA *UCA1* enhanced EZH2 expression, which subsequently elevated the expression of cyclin D1 to promote cell cycle [50]. These findings supported the previous hypothesis that the association of lncRNA *UCA1* in regulating other genes via epigenetic modification, which is histone modification in this case. The association of lncRNA *UCA1* with AKT/GSK-3B/cyclin D1 was also identified in HCC [60].

In addition to EMT, lncRNA *UCA1* can induce GC metastasis by regulating G protein-coupled receptor kinase 2 (GRK2) degradation and Casitas B-lineage Lymphoma (Cbl-c)-mediated ubiquitination, resulting in the activation of extracellular-signal-regulated kinase (ERK)/matrix metalloproteinase-9 (MMP-9) signaling pathway [51]. Wang et al. demonstrated that lncRNA *UCA1* interacted with GRK2 and led to the exposure of GRK2 ubiquitination sites toward Cbl-c for its degradation [51]. Consequently, the degraded GRK2 activated ERK/MMP-9 signaling pathway, which increased MMP-9 protein levels, to promote cell membrane degradation, facilitating cancer cell migration and invasion [51]. This finding showed that lncRNA *UCA1* could regulate the level of another protein by direct binding for degradation.

LncRNA *UCA1* also plays a prominent role in chemoresistance via miRNA signaling. For instance, the silenced lncRNA *UCA1* could upregulate the mRNA levels of *miR-27b* and lead to reduced IC_50_ of doxorubicin, cisplatin, and 5-fluorouracil, as well as promoting doxorubicin-induced apoptosis in doxorubicin-resistance SGC-7901 GC cells [52]. In other words, the reduction of lncRNA *UCA1* expression could improve the chemosensitivity of chemotherapeutic agents, at least for doxorubicin, cisplatin, and 5-fluorouracil in GC therapy. Correspondingly, Cheng et al. reported that lncRNA *UCA1* silencing enhanced GC chemosensitivity toward cisplatin by regulating the expression of miR-513a-3p and Cytochrome P450 1B1 (CYP1B1) [53].

Chemoresistance is also affected by cancer microenvironment, such as hypoxic microenvironment, that claims to block the exposure of chemotherapeutic agents to cancer cells [54]. Yang et al. reported that GC cells could survive in the hypoxic environment via the interaction of lncRNA *UCA1* with miR-7-5p, elevating the expression of epidermal growth factor receptor (EGFR) in hypoxia-resistant GC cells [54]. Nonetheless, chronic hypoxia environment with a slight increment in the protein levels of hypoxia-inducible factor-1alpha (HIF-1*α*) could reduce lncRNA *UCA1* expression [54]. Taken together, these findings demonstrated that the lncRNA *UCA1* may facilitate GC development, progression, and chemoresistance via the interaction with different molecules, signaling pathways, and/or miRNAs.

### 3.3. Hepatobiliary Cancer

Hepatobiliary cancer comprises tumors present in the liver, gallbladder, and bile duct (cholangiocarcinoma). For instance, Wang et al. showed that lncRNA *UCA1* was highly expressed in HCC and positively correlated with postoperative survival and tumor, node, and metastasis (TNM) stage [78]. In addition, the result also showed that lncRNA *UCA1* regulated fibroblast growth factor receptor 1 (FGFR1)/ERK signaling pathway through sponging miR-216b that led to downregulation of the mRNA levels of *miR-216b*. In contrast, upregulation was detected for *fgfr1* gene to activate the ERK signaling pathway [78].

One of the known risk factors for HCC is hepatitis virus infection [79]. Interestingly, hepatitis B virus (HBV) can induce lncRNA *UCA1* in HCC cells via their produced X protein (HBx) [55]. LncRNA *UCA1* also significantly reduced p27kip1 expression along with the increased expression of EZH2 via histone methylation on p27kip1 promoter region [55]. In addition, ectopically expressed lncRNA *UCA1* induced the expression of cyclin-dependent kinase-2 (CDK2) but not for CDK4 and CDK6 where CDK2 regulated cell cycle and apoptosis, and its activity was regulated by CDK inhibitors (e.g., p21 and p27) [55]. However, only p27 expression was suppressed in overexpressed HBx and lncRNA *UCA1* HCC cells [55]. Therefore, this finding suggested that the regulating effects of lncRNA *UCA1* are protein-specific despite originating from the same upstream mediators.

Apart from lncRNA *UCA1*, TGF-*β*1 and HXK2 were also found to be overexpressed in HCC patients [56]. Hu et al. suggested that TGF-*β*1 promoted HCC cell growth through the induction of energy metabolism and subsequently promoted lncRNA *UCA1* expression and its downstream regulator HXK2, an isozyme that involves in glycolysis [56]. Most studies have reported that lncRNA *UCA1* is prone to regulate miRNA expression, but Zhao et al. revealed that *miR-124*, a tumor suppressor mRNA, reduced rho-associated protein kinase 1 (ROCK1) to suppress lncRNA *UCA1* expression, leading to the inhibition of HCC cell proliferation, migration, and invasion [57]. They further discovered that the expression of both lncRNA *UCA1* and miR-124 was not affected by HBV and HCV infections [57]. This finding, however, could be correct if lncRNA *UCA1* is the downstream target protein of miR-124 or incorrect if miRNA and lncRNA *UCA1* are negatively regulated in which miRNAs usually downregulated when lncRNA *UCA1* is overexpressed as in most cancer types reported.

Furthermore, staphylococcal nuclease and tudor domain containing 1 (SND1) can induce the expression of lncRNA *UCA1* through its interaction with myeloblastosis proto-oncogene (MYB), a transcriptional activator, by forming SND1-MYB complex [58]. Meanwhile, SND1 itself acts as an antiapoptotic factor in HCC [58]. Again, this finding supported the previous hypothesis that lncRNA *UCA1* expression can be induced by another gene or protein.

Meanwhile, an *in vitro* study involving HCC cells showed that lncRNA *UCA1* was substantially induced by arsenic (As) at 10 *μ*M/L with > 4-fold increase, denoting a protective role against As-induced cell death [59]. By using RNA-Seq assay, oxidative stress induced growth inhibitor 1 (OSGIN1) was uncovered to be the most responsive downstream target of lncRNA *UCA1*, and miR-184 acted as an intermediate for the regulation of lncRNA *UCA1* on OSGIN1 expression through ceRNA mechanism [59]. The lncRNA *UCA1*/OSGIN1 signaling contributed to As-induced autophagic flux blockage through activating mTOR/ribosomal protein S6 kinase beta-1 (p70S6K) cascade and therefore resulting in compromised cell death [59]. Nonetheless, this finding did not directly conclude the relationship of lncRNA *UCA1* with HCC progression. However, the arsenic stress might resemble anticytotoxicity effects as arsenic induces cell death. Therefore, future studies should be conducted in order to relate the effects of lncRNA *UCA1*/OSGIN1/mTOR/p70S6K with HCC progression.

On the other hand, overexpressed lncRNA *UCA1* in cholangiocarcinoma (CCA) showed that it could act as an independent prognostic factor in CCA patients [60]. Similar to the finding reported by Wang et al. in GC, Xu et al. also found that enhanced CCA cell proliferation was via the activation of AKT/GSK-3*β* axis that led to upregulation of cyclin D1 (CCND1) expression [50, 60]. The apoptosis inhibition in highly lncRNA *UCA1*-expressed CCA cells might be partly due to B-cell lymphoma 2 (Bcl-2)/caspase-3 pathway [60].

LncRNA *UCA1* has also been reported to play an important role in CCA metastasis through regulating miR-122/chloride intracellular channel 1 (CLIC1). For instance, both lncRNA *UCA1* and CLIC1 were elevated, while miR-122 was reduced in bile duct carcinoma [61]. Also, both lncRNA *UCA1* and CLIC1 promoted the phosphorylation of ERK and mitogen-activated protein kinase (MAPK), activating ERK/MAPK signaling pathway to promote cancer cell metastasis [61].

Apart from HCC and CCA, lncRNA *UCA1* is also overexpressed in gallbladder cancer (GBC) [62]. The overexpressed lncRNA *UCA1* regulated tumor progression through the recruitment of EZH2 to the promoter of both tumor suppressor p21 and E-cadherin that resulted in their suppressed expression [62]. This observation is opposed to what discovered in HCC by Hu et al. for p21, which could be probably explained by different cancer types used.

In short, these findings revealed the association of lncRNA *UCA1* in tumor progression, invasion, and metastasis of hepatobiliary cancer by regulating downstream molecules or be regulated by upstream mediators.

### 3.4. Pancreatic Cancer

Pancreatic cancer (PC) is the fourth leading cause of cancer-related deaths worldwide [80, 81]. According to Chen et al., lncRNA *UCA1* overexpression was detected in the tissues of 128 pancreatic cancer patients compared to adjacent nontumor tissues [63]. Moreover, lncRNA *UCA1* silencing inhibited cell proliferation and induced apoptosis and cell cycle arrest in PC cells [63]. They also found the possible association of lncRNA *UCA1* with the inhibition of p27 in their previous study on PC [63]. In addition, lncRNA *UCA1* was shown to regulate growth and metastasis by sponging miR-135a in PC [64]. Apart from the interaction with miR-135a, lncRNA *UCA1* also inhibited *miR-96*, a tumor suppressor mRNA, resulting in the upregulation of forkhead box O-3 (FOXO3) to promote tumor progression [65].

In PC cells, lncRNA *UCA1* demonstrated to promote cell migration and invasion through Hippo pathway by interacting with key proteins, such as Mps one binder kinase activator (MOB1), large tumor suppressor kinase 1 (Lats1), phosphorylated-Lats1, and Yes-associated protein (YAP) [66]. LncRNA *UCA1* bound to MOB1, Lats1, and YAP to form three shielding composites, retaining YAP activation and leading to YAP translocation into the nucleus to induce gene expression for cell proliferation and apoptosis and for lncRNA *UCA1* expression itself [66]. Moreover, lncRNA *UCA1* also interacted with MOB1, Lats1, and YAP to form ribonucleoprotein complex that could be another reason in regulating gene expression. In addition, upregulation of MMP (e.g., MMP14, MMP2, and MMP9) proteins were also detected in lncRNA *UCA1*-overexpressed PC cells, suggesting the role of lncRNA *UCA1* in invasion and migration [66]. This study indicated that lncRNA *UCA1* could interact with key proteins and protein complexes by binding to their promoter region to enhance PC cell progression.

In pancreatic ductal adenocarcinoma (PDAC), lncRNA *UCA1* regulated miR-590-3p to increase the expression of oncogenic Kirsten rat sarcoma viral oncogene homolog (KRAS) protein, and KRAS itself can promote lncRNA *UCA1* expression [67]. This discovery showed that lncRNA *UCA1* and its downstream protein could regulate each other. Previously, Gu et al. reported that lncRNA *UCA1* was associated with miR-590-3p in GC cells via the target gene of miR-590-3p and *creb1* [46]. Interestingly, Liu et al. newly discovered that *kras* is another target gene of miR-590-3p in PDAC [67]. Therefore, further studies could be conducted to identify miRNA target genes associated with lncRNA *UCA1* to enhance the understanding of the exact mechanism in regulating PDAC progression.

Interesting observation by using human PDAC PANC-1 cells showed the potential of ceRNA networks, consisting of lncRNAs, circRNAs, and mRNAs, to be involved in autophagy suppression of PDAC caused by chloroquine diphosphate [82]. By using microarrays, numerous ceRNAs exhibited target associations with miR-663a-5p and miR-154-3p, and negative associations with the expression of the targeted miRNAs under the same changes in the autophagic level were determined [82]. The study also demonstrated that AC024560.2 competitively binds to miR-663a-5p and thus regulates the autophagic level of PDAC cells by inhibiting the expression of this miRNA [82]. This shows that the ceRNAs including lncRNA could be a potential molecular target in diagnosis and treatment of PC.

To sum up, lncRNA *UCA1* plays a significant role in PC progression that could be a novel independent predictor of the poor survival of PC patients, as well as a promising biomarker in cancer therapy.

### 3.5. Colorectal Cancer

Highly expressed lncRNA *UCA1* is also reported in colorectal cancer (CRC) cells and contributed to tumorigenic activity [68]. For instance, overexpressed lncRNA *UCA1* reduced miR-204-5p expression in CRC cells to enhance the expression of miR-204-5p target proteins, such as BCL2, ras-related protein (RAB22A), and CREB1 [69]. Elevated expression of BCL2 and RAB22A can promote CRC cell proliferation and drug resistance, while CREB1 transcription factor involves in CRC tumorigenesis [69, 83]. In addition to miR-204-5p, *creb1* is also a target gene of miR-590-3p [46].

Similarly, lncRNA *UCA1* also inhibited miR-28-5p activity to cause the overexpression of Homeobox B3 (HOXB3), promoting CRC cell proliferation and migration [70]. Cui et al. revealed that both lncRNA *UCA1* silencing and elevation of miR-28-5p expression reduced the protein levels of MMP2 and MMP9 that play a crucial role in cancer cell metastasis [70].

Interestingly, lncRNA *UCA1* also has an miRNA sponging activity in CRC. For instance, it sponged miR-185-5p and led to overexpressed Wnt family member 1 (WNT1) and WNT1-inducible-signaling pathway protein 2 (WISP2); both activating WISP2/*β*-catenin signaling pathway to regulate autophagy and survival of CRC [71]. Apart from *wnt1* and *wisp2*, *mapk14* is also a target gene of miR-185-5p, where upregulation of *mapk14* activated mitogen-activated protein kinase-activated protein kinase 2 (MAPKAPK2)/heat-shock protein 27 (HSP27) signaling pathway to promote invasion, migration, and EMT [72].

The interplay of CRC tumor microenvironment on the expression of lncRNA *UCA1* has also been studied. Jahangiri et al. demonstrated that cancer-associated fibroblasts (CAFs) activated lncRNA *UCA1* to induce mTOR overexpression [73]. The active lncRNA *UCA1*/mTOR axis subsequently reduced the expression of tumor suppressor p27 and miR-143 while significantly increased cyclin D1 and KRAS expression [73]. Nonetheless, they further discovered that mTOR can regulate miR-143, but whether lncRNA *UCA1* could directly regulate the expression of miR-143 is unknown.

Interestingly, it was discovered that the expression of lncRNA *UCA1* was significantly higher in four CRC human tissues and CCL244 CRC cells, but no significant difference was observed in HCT-116 CRC cells after chemoradiotherapy [74]. This observation may indicate that lncRNA *UCA1* plays a regulatory role in CRC radioresistance. Nevertheless, when lncRNA *UCA1* was silenced, it enhanced the radiotherapy sensitivity of CRC cells via X-ray irradiation-induced apoptosis and prolonged G2/M cell cycle [74]. Yang et al. further showed that low level of lncRNA *UCA1* inhibited EMT induction by significantly suppressing the expression of EMT-regulating proteins, such as MMP2, MMP9, ZEB1, and vimentin [74]. In addition, the regulation of lncRNA *UCA1* in CRC chemoresistance is also facilitated by autophagy. For instance, it was shown to promote 5-fluorouracil resistance in CRC cells by facilitating autophagy mediated by repressed miR-23b-3p and elevated zinc finger protein 281 (ZNF281) [75]. Similarly, lncRNA *UCA1* also mediated autophagy to protect BC against rapamycin by inducing miR-582-5p-regulated autophagy-related protein 7 (ATG7) [84].

Meanwhile, Yang et al. illustrated that exosomal lncRNA *UCA1* could be a promising biomarker for effective diagnosis and targeted therapy as exosomal lncRNA *UCA1* can be assayed in a noninvasive manner and found to be relatively abundant and stable in the serum of CRC patients [76]. To note, exosomes originated from cetuximab-resistance cell can alter the expression of lncRNA *UCA1* and enhance resistance to cetuximab in CRC cells in view of the fact that lncRNA *UCA1* can transmit cetuximab resistance to sensitive cells [76]. Given this circumstance, exosomal lncRNA *UCA1* indeed has a great potential to be used as an evaluation factor for predicting cetuximab chemoresistance in CRC patients.

In summary, lncRNA *UCA1* participated significantly in the CRC progression, invasion, migration, metastasis, radioresistance, and chemoresistance. Therefore, lncRNA *UCA1* can be a promising molecular target to combat CRC in chemotherapy, as well as in diagnostic and prognostic purpose of CRC patients.

## 4. Conclusion

This review has provided an insight into the regulatory roles and patterns of lncRNA *UCA1* in GI cancer progression and chemoresistance, as well as its underlying mechanisms and interaction with key molecules involved, which may serve as a novel and highly potential molecular target for GI cancer therapy. It has discovered that multiple preclinical and clinical studies supporting the oncogenic role of lncRNA *UCA1* in GI cancer. In addition, the potential of lncRNA *UCA1* to be used as a prognostic marker has also been reported in several studies, where its expression correlates with the TNM stage of GI cancer [85]. Based on the findings in this review, it was revealed that basic overexpression of lncRNA *UCA1* has a positive implication in initiation, proliferation, invasion, migration, and chemoresistance of GI cancer, although contradictory findings claim that it also has anticancer activity, via the interactions with upstream and/or downstream molecules, signaling pathways, or biological processes. The regulatory roles of lncRNA *UCA1* in GI cancer progression are relatively observed more in GC followed by CRC. Comparatively, the regulation of chemoresistance by lncRNA *UCA1* has so far discovered only in GC and CRC [16]. In general, lncRNA *UCA1* interacts with miRNAs, leading to the reduction of its target gene expression, such as sponging miR-185-5p, in CRC. Moreover, a similar miRNA sponging activity by lncRNA *UCA1* can be observed in different GI cancers, such as miR-590-3p in GC and PDAC [46, 67]. LncRNA *UCA1* also modulates several gene expressions through epigenetic regulation, particularly associated with histone and chromatin modifications. For instance, lncRNA *UCA1* interacts with EZH2 to induce histone methylation as observed in GC, HCC, and CCA [50, 55, 62].

The strategy of lncRNA *UCA1* silencing conducted by many researchers showed a promising result in combating GI cancer progression and chemoresistance. Moreover, targeted therapies against lncRNA *UCA1* can also be developed for cancer therapy. The approaches that could be taken to achieve this purpose include lncRNA *UCA1* silencing via RNA interference (RNAi) and structural disruption of lncRNA [86, 87]. In addition, the research of active compounds from the natural products, particularly plants, also could be considered in order to achieve this purpose. This is because the active phytochemicals in many herbal plants have shown to exert potent cytotoxic effects against various cancers, including GI cancer [88–90]. In conclusion, lncRNA *UCA1* has been identified as a novel and potential molecular target for GI cancer in the last decade based on its potent regulatory roles in cancer progression and chemoresistance. However, to enhance its translation possibility to clinical trials, more preclinical studies using both *in vitro* and *in vivo* models should be conducted to further explore the key mechanism of actions underlying its regulatory roles. Also, lncRNA *UCA1*, particularly enriched in exosomes, can be a potential diagnostic and prognostic biomarker compared to other molecular targets due to its high stability and availability in various human body fluids, including urine for BC [13], serum for HCC [91], and plasma sample in early GC [42], as well as its possible simplicity of extraction and diagnostic testing procedures.

## Figures and Tables

**Table 1:** Summary of the studies that assessed the expression and regulatory roles of lncRNA *UCA1* in human cell lines and tissues of GI cancer.

Cancer type	Study subject	Cell line	Finding/mechanistic response	Reference
Esophageal cancer	90 ESCC patients who underwent surgery	EC109, EC9706, KYSE150, KYSE510, and NE1	(i) LncRNA *UCA1* was overexpressed and contributed to poor prognosis	[37]
			(ii) Silenced lncRNA *UCA1* decreased cell proliferation, migration, and invasion	
	66 esophageal cancer patients underwent surgical resection	EC9706 and KYSE	(i) LncRNA *UCA1* was overexpressed and contributed to poor prognosis	[38]
			(ii) *Sox4* was identified as a direct target gene of lncRNA *UCA1* and acted as a ceRNA	
			(iii) LncRNA *UCA1* reduced miR-204 level	
	110 EC tissues and 60 paired of adjacent nontumorous tissues	EC1, EC109, EC9706, KYSE150, and Het-1A	(i) LncRNA *UCA1* was overexpressed in EC tissues with advanced EC stages and was associated with poor prognosis	[39]
			(ii) Overexpressed lncRNA *UCA1* promoted cell proliferation and metastasis	
			(iii) LncRNA *UCA1* promoted glycolysis by sequestering miR-203 to increase HK2 levels, resulting in enhanced Warburg effect	
	106 newly diagnosed patients with primary cancer and previously untreated ESCC	EC109	(i) LncRNA *UCA1* lowly expressed in tumor tissue compared to the adjacent nontumor tissue	[40]
			(ii) LncRNA *UCA1* suppressed ESCC via inhibition of Wnt signaling pathway	
			(iii) LncRNA *UCA1* reduced C-myc and active *β*-catenin protein expression	
	15 paired EC tissues and adjacent normal tissues of EC patients	EC18, KYSE140, and NEEC	(i) LncRNA *UCA1* expression was decreased in EC tissues and plasma exosomes	[41]
			(ii) LncRNA *UCA1* inhibited cell proliferation, invasion, migration, and colony formation as well as inhibited tumor growth *in vivo*	
			(iii) Exosomal lncRNA *UCA1* directly targeted miRNA-613 in EC cells	
Gastric cancer	20 plasma samples of patients and pair-matched plasma samples	Five GC tissues and five pair-matched noncancerous tissues	(i) Overexpressed lncRNA *UCA1* in both GC tissue and plasma of GC patients	[42]
			(ii) Plasma lncRNA *UCA1* provided higher diagnostic performance for the detection of GC	
Gastric cancer	112 patients diagnosed with GC	SGC-7901, BGC-823, MKN-28, AGS, and GES-1	(i) Overexpressed lncRNA *UCA1* in GC human tissue and GC cell lines	[43]
			(ii) High lncRNA *UCA1* expression correlated with worse differentiation, tumor size, invasion depth, and TNM stage	
	Chinese patients	BGC-823 and SGC-7901	(i) Elevated lncRNA *UCA1* in tumor tissues of GC patients	[44]
			(ii) LncRNA *UCA1* promoted metastasis by sponging miR-203, resulting in ZEB overexpression	
	Ten GC and ten paracancerous normal tissues from the patients in China	MGC‐803, SGC‐7901, BGC‐823, AGS, MKN‐45, and GES‐1.	(i) LncRNA *UCA1* expression was higher in GC compared to paracancerous tissues	[45]
			(ii) SATB1 and lncRNA *UCA1* competitively bound to miR‐495‐3p that acts as a ceRNA and reduced its expression	
	62 GC patients who underwent surgical resection	AGS, MKN-28, SGC-7901, MKN-45, and GES-1	(i) Overexpressed lncRNA *UCA1* in GC human tissue and GC cell lines	[46]
			(ii) LncRNA *UCA1* repressed miR-590-3p, leading to increased CREB1 expression	
	40 primary GC tissues and corresponding adjacent nontumorous gastric tissue samples	AGS, SGC-7901, BGC-823, MGC-803, and SNU-1	(i) Overexpressed lncRNA *UCA1* in GC human tissue compared to adjacent noncancerous tissues	[47]
			(ii) LncRNA *UCA1* repressed miR-26a/b, miR-193a, and miR-214 expression through direct interaction	
			(iii) LncRNA *UCA1* upregulated *pdl1*	
	37 paired GC tissues and corresponding adjacent normal tissues	HGC27, MGC803, NCI-N87, BGC-823, SGC7901, and GES-1	(i) Overexpressed lncRNA *UCA1* in GC human tissue compared to adjacent normal tissues	[48]
			(ii) TGFb1-induced lncRNA *UCA1* elevation and acceleration of EMT	
	102 gastric cancer patients who underwent surgery	MKN-28, SGC-7901, MGC-803, BGC-823, MKN-45, and GES-1	(i) The overexpression of *UCA1* in GC was higher in GC tissue than adjacent noncancerous tissues, and it is correlated with TNM stage and lymph node metastases	[49]
			(ii) LncRNA *UCA1* activated PI3K-Akt-mTOR signaling pathway	
	39 patients with GC	BGC-823, SGC-7901, AGS, MKN-45, NCI-N87, and MKN-28	(i) LncRNA *UCA1* highly expressed in GC tissues than its matched nontumor tissues	[50]
			(ii) SP1 induced lncRNA *UCA1*	
			(iii) EZH2 and lncRNA *UCA1* interaction activated AKT/GSK-3B/cyclin D1 axis	
	49 patients with GC	MGC-803, HGC-27, NCI-N87, and GES-1	(i) LncRNA *UCA1* was highly expressed in GC tissues than its adjacent nontumor tissues	[51]
			(ii) LncRNA *UCA1* promoted tumor metastasis by inducing GRK2 degradation, which activated the ERK-MMP9 signaling pathway	
	28 primary GC patients who had not received previous chemotherapy or radiotherapy	SGC-7901, SGC-7901, SGC-7901/ADR, SGC-7901/DDP, and SGC-7901/FU	(i) LncRNA *UCA1* was one of the lncRNAs overexpressed in GC tissue	[52]
			(ii) Multidrug resistance of GC by repressing miR-27b	
	53 pairs of GC tissues and adjacent normal tissues	GES-1, SNU-5, AGS, and NCI-N87	(i) LncRNA *UCA1* was highly expressed in GC tissues than its adjacent nontumor tissues	[53]
			(ii) Knockdown of lncRNA *UCA1* increased sensitivity to cisplatin by inducing cell apoptosis	
			(iii) LncRNA *UCA1* reduced miR-513a-3p and elevated CYP1B1	
	—	MGC-803 and BGC-823	(i) LncRNA *UCA1* promoted the migration of hypoxia-resistant GC cells via miR-7-5p/EGFR axis	[54]
Hepatobiliary cancer	60 paired tumorous and adjacent nontumorous liver tissues obtained immediately after surgical resection	LO2 cells and HBx-expressing hepatoma cells	(i) HBx induced lncRNA *UCA1* expression in hepatocytes	[55]
			(ii) LncRNA *UCA1* reduced p27kip1 expression and increased EZH2 expression via histone methylation on p27kip1 promoter region	
			(iii) LncRNA *UCA1* induced CDK2 expression without altering CDK4 and CDK6	
	88 HCC patients	HepG2 and Huh7	(i) LncRNA *UCA1* highly expressed in 79 patients out of 88 HCC patients	[56]
			(ii) TGF-*β*1 induced the expression of lncRNA *UCA1* and HXK2	
	66 newly diagnosed HCC patients	SNU-398 and SNU-449	(i) Overexpressed lncRNA *UCA1* was detected in HCC tissues compared to healthy tissues	[57]
			(ii) miR-124 repressed ROCK1	
			(iii) ROCK1 reduced lncRNA *UCA1* expression	
			(iv) HBV and HCV infections did not affect the expression of lncRNA *UCA1* and miR-124	
	50 HCC patients from online data sets	HEK 293t and HepG2	(i) Overexpressed SND1 in HCC tissues than normal tissues	[58]
			(ii) SND1 induced lncRNA *UCA1* expression through the interaction of SND1 with MYB	
	—	HepG2	(i) Arsenic stress induced lncRNA *UCA1*	[59]
			(ii) LncRNA *UCA1* promoted protective roles of arsenic-induced cell death by blocking autophagic flux	
			(iii) LncRNA *UCA1* protected HCC cells against arsenic stress by repressing miR-184 and elevating OSGIN1 that activated mTOR/p70S6K autophagy inhibition pathway	
	68 CCA patients	HCCC-9810, RBE, QBC939, Huh-28, HuCCT1, KMBC, CCLP-1, and HIBEC	(i) LncRNA *UCA1* was overexpressed in CCA tissues and cell lines	[60]
			(ii) LncRNA *UCA1* inhibited apoptosis through Bcl-2/caspase-3 pathway	
			(iii) Activated AKT/GSK-3*β* axis elevated CCND1 expression	
	22 CCA patients receiving surgical resection	LIPF155C, CCLP1, QBC939, huh28, and HIBEC	(i) LncRNA *UCA1* was highly expressed lncRNA in CCA compared with paracarcinoma tissues	[61]
			(ii) Regulation of miR-122/CLIC1 and activation of ERK/MAPK signaling pathway	
	45 GBC tissues and neighboring noncancerous tissues from patients who underwent liver resection	NOZ and GBC-SD	(i) High expression of lncRNA *UCA1* was associated with tumor size, lymph node metastasis, TNM stage, and short survival time in GBC patients	[62]
			(ii) Recruitment of EZH2 to the promoter of p21 and E-cadherin	
			(iii) Epigenetically suppressed p21 and E-cadherin expression	
Pancreatic cancer	128 PC patients received operation as initial systemic treatment	Panc-1, Bxpc-3, Capan-1,SW-1990, and HPDE6C-7	(i) LncRNA *UCA1* overexpressed in PC tissue and cell lines	[63]
			(ii) LncRNA *UCA1* suppressed p27 protein	
	50 PC patients	SW1990, BxPC-3, MiaPaCa-2, PANC-1, CAPAN-1, and HPDE	(iii) Highly expressed lncRNA *UCA1* in PC tissues and cell lines	[64]
			(iv) LncRNA *UCA1* sponged miR135a	
	36 PC patients underwent surgical resection	HPC-Y5, PANC-1, SW1990, and AsPC-1	(i) Out of 19 lncRNAs, lncRNA *UCA1* was one of the overexpressed lncRNAs in PC tissues	[65]
			(ii) LncRNA *UCA1* repressed miR-96, resulting in increased FOXO3 expression	
	Analysis of mRNA levels of lncRNA *UCA1* in PC patients from BADEA and TCGA databases	BxPC-3, SW1990, PaTu8988, and PANC-1	(i) Higher mRNA levels of lncRNA *UCA1* in PC tissues than normal pancreatic tissues and correlated with poor prognosis	[66]
			(ii) LncRNA *UCA1* promoted cell migration and invasion via Hippo signaling pathway	
	Analysis of lncRNA *UCA1* mRNA levels from TCGA database in PDAC tumor specimens and normal	PaTu8902, Mpanc96, HEK293T, and H6C7	(i) LncRNA *UCA1* was highly expressed in PDAC tumor specimens than normal tissue	[67]
			(ii) LncRNA *UCA1* acted as a ceRNA to increase the expression of KRAS via sponging miR-590-3p	
			(iii) KRAS promoted lncRNA *UCA1* expression.	
Colorectal cancer	80 CRC patients	CaCO-2, SW480, HCT116, LoVo, and CCC-HIE-2	(i) Overexpressed lncRNA *UCA1* promoted cell proliferation, apoptosis, and cell cycle distribution	[68]
	Two CRC cohorts, including 90 and 119 human primary CRC tissues and their paired adjacent noncancerous tissues, respectively	HEK-293T, HCT8, HCT116, HT29, LoVo, and SW480	(i) Induced 5-FU resistance	[69]
			(ii) Inhibition of miR-204-5p and upregulated its target genes (e.g., *bcl2, rab22a, and creb1)*	
	60 CRC patients	NCM460, SW620, HT29, CACO2, SW480, and HCT116	(i) Overexpressed lncRNA *UCA1* in CRC tissues and cell lines	[70]
			(ii) LncRNA *UCA1* repressed miR‐28‐5p level, which subsequently increased HOXB3 axis	
			(iii) LncRNA *UCA1* elevated MMP2 and MMP9	
	—	CCD-18Co, HIEC-6, SW620, and HT29	(i) Overexpressed lncRNA *UCA1* in CRC cell lines	[71]
			(ii) LncRNA *UCA1* sponged miR-185-5p, leading to elevation of WNT1 and WISP2 that activated WISP2/b-catenin signaling pathway, which affected autophagy and survival of CRC	
	—	SW480, SW620, HT-29, CCD-18Co, and HIEC-6	(i) Overexpressed lncRNA *UCA1* in CRC cell lines	[72]
			(ii) LncRNA *UCA1* elevated the expression of MAPK14 to activate MAPKAPK2/HSP27 signaling pathway	
	—	SW480 and NF	(i) Overexpressed lncRNA *UCA1* in CRC cell lines	[73]
			(ii) CAFs induced lncRNA *UCA1* to increase the expression of mTOR	
			(iii) LncRNA *UCA1*/mTOR axis repressed p27 and miR-143 and significantly elevated cyclin D1 and KRAS expression	
	Tissue from 32 CRC patients collected immediately after surgical resection	HCT116, CCL244, SW480, LoVo, and FHC	(i) LncRNA *UCA1* significantly expressed higher in CRC tissue after chemoradiotherapy	[74]
			(ii) Downregulation of LncRNA *UCA1* enhanced radiotherapy sensitivity	
			(iii) LncRNA *UCA1* inhibited EMT by reducing MMP2, MMP9, ZEB1, and vimentin	
	25 CRC patients with 5-fluorouracil resistance and 25 CRC patients with 5-fluorouracil sensitivity	SW480, SW620, and 293T	(i) 5-fluorouracil resistance of CRC was associated with lncRNA *UCA1* abundance that promoted autophagy and inhibited apoptosis	[75]
			(ii) LncRNA *UCA1* sponged miR-23b-3p and consequently elevated ZNF281 expression	
	53 CRC patients treated with cetuximab	Caco2-CR and Caco2-CS	(i) LncRNA *UCA1* levels upregulated in cetuximab-resistant cells and their exosomes	[76]
			(ii) Exosomal lncRNA *UCA1* was detectable and stable in the serum of CRC patients	
			(ii) Exosomes originated from cetuximab-resistant cells could alter lncRNA *UCA1* expression	
			(iv) LncRNA *UCA1* can be transferred from resistant cells to sensitive cells through exosomes	

## Data Availability

The data supporting this manuscript are extracted from the previously reported studies and data sets, which have all been cited.

## References

[1] Hahne J. C., Nicola V. (2018). Non-coding RNAs and resistance to anticancer drugs in gastrointestinal tumors. *Frontiers in Oncology*.

[2] Pourhoseingholi M. A., Vahedi M., Baghestani A. R. (2015). Burden of gastrointestinal cancer in Asia; an overview. *Gastroenterology and Hepatology from Bed to Bench*.

[3] Arnold M., Abnet C. C., Neale R. E. (2020). Global burden of 5 major types of gastrointestinal cancer. *Gastroenterology*.

[4] Bray F., Ferlay J., Soerjomataram I., Siegel R. L., Torre L. A., Jemal A. (2018). Global cancer statistics 2018: GLOBOCAN estimates of incidence and mortality worldwide for 36 cancers in 185 countries. *CA: A Cancer Journal for Clinicians*.

[5] Allemani C., Matsuda T., Di Carlo V. (2018). Global surveillance of trends in cancer survival 2000–14 (CONCORD-3): analysis of individual records for 37 513 025 patients diagnosed with one of 18 cancers from 322 population-based registries in 71 countries. *The Lancet*.

[6] Ringehan M., McKeating J. A., Protzer U. (2017). Viral hepatitis and liver cancer. *Philosophical Transactions of the Royal Society B: Biological Sciences*.

[7] Wroblewski L. E., Peek R. M., Wilson K. T. (2010). *Helicobacter pylori* and gastric cancer: factors that modulate disease risk. *Clinical Microbiology Reviews*.

[8] Tang Y., Cheung B. B., Atmadibrata B. (2017). The regulatory role of long noncoding RNAs in cancer. *Cancer Letters*.

[9] Uszczynska-Ratajczak B., Lagarde J., Frankish A., Guigó R., Johnson R. (2018). Towards a complete map of the human long non-coding RNA transcriptome. *Nature Reviews Genetics*.

[10] Zampetaki A., Albrecht A., Steinhofel K. (2018). Long non-coding RNA structure and function: is there a link?. *Frontiers in Physiology*.

[11] Hocine S., Singer R. H., Grünwald D. (2010). RNA processing and export. *Cold Spring Harbor Perspectives in Biology*.

[12] Schmitt A. M., Chang H. Y. (2016). Long noncoding RNAs in cancer pathways. *Cancer Cell*.

[13] Wang X.-S., Zhang Z., Wang H.-C. (2006). Rapid identification of UCA1 as a very sensitive and specific unique marker for human bladder carcinoma. *Clinical Cancer Research*.

[14] Ghafouri-Fard S., Taheri M. (2019). UCA1 long non-coding RNA: an update on its roles in malignant behavior of cancers. *Biomedicine & Pharmacotherapy*.

[15] Tsang W. P., Wong T. W. L., Cheung A. H. H., Co C. N. N., Kwok T. T. (2007). Induction of drug resistance and transformation in human cancer cells by the noncoding RNA CUDR. *RNA*.

[16] Wang H., Guan Z., He K., Qian J., Cao J., Teng L. (2017). LncRNA UCA1 in anti-cancer drug resistance. *Oncotarget*.

[17] Xue M., Chen W., Li X. (2016). Urothelial cancer associated 1: a long noncoding RNA with a crucial role in cancer. *Journal of Cancer Research and Clinical Oncology*.

[18] Yao F., Wang Q., Wu Q. (2019). The prognostic value and mechanisms of lncRNA UCA1 in human cancer. *Cancer Management and Research*.

[19] Hosseini N. F., Manoochehri H., Khoei S. G. (2021). The functional role of long non-coding RNA UCA1 in human multiple cancers: a review study. *Current Molecular Medicine*.

[20] Wang Z., Gerstein M., Snyder M. (2009). RNA-Seq: a revolutionary tool for transcriptomics. *Nature Reviews Genetics*.

[21] Jarroux J., Morillon A., Pinskaya M. (2017). History, discovery, and classification of lncRNAs. *Long Non Coding RNA Biology*.

[22] Dahariya S., Paddibhatla I., Kumar S., Raghuwanshi S., Pallepati A., Gutti R. K. (2019). Long non-coding RNA: classification, biogenesis and functions in blood cells. *Molecular Immunology*.

[23] Fatica A., Bozzoni I. (2014). Long non-coding RNAs: new players in cell differentiation and development. *Nature Reviews Genetics*.

[24] Schmitz S. U., Grote P., Herrmann B. G. (2016). Mechanisms of long noncoding RNA function in development and disease. *Cellular and Molecular Life Sciences*.

[25] Quinn J. J., Chang H. Y. (2016). Unique features of long non-coding RNA biogenesis and function. *Nature Reviews Genetics*.

[26] Balas M. M., Johnson A. M. (2018). Exploring the mechanisms behind long noncoding RNAs and cancer. *Non-coding RNA Research*.

[27] Neve B., Jonckheere N., Vincent A., Van Seuningen I. (2018). Epigenetic regulation by lncRNAs: an overview focused on UCA1 in colorectal cancer. *Cancers*.

[28] Sawyer I. A., Dundr M. (2017). Chromatin loops and causality loops: the influence of RNA upon spatial nuclear architecture. *Chromosoma*.

[29] Kawaguchi T., Hirose T. (2015). Chromatin remodeling complexes in the assembly of long noncoding RNA-dependent nuclear bodies. *Nucleus*.

[30] West J. A., Davis C. P., Sunwoo H. (2014). The long noncoding RNAs NEAT1 and MALAT1 bind active chromatin sites. *Molecular Cell*.

[31] Wang K. C., Chang H. Y. (2011). Molecular mechanisms of long noncoding RNAs. *Molecular Cell*.

[32] Morlando M., Fatica A. (2018). Alteration of epigenetic regulation by long noncoding RNAs in cancer. *International Journal of Molecular Sciences*.

[33] Paraskevopoulou M. D., Hatzigeorgiou A. G. (2016). Analyzing miRNA–lncRNA interactions. *Long Non-coding RNAs*.

[34] Wang F., Li X., Xie X., Zhao L., Chen W. (2008). UCA1, a non-protein-coding RNA up-regulated in bladder carcinoma and embryo, influencing cell growth and promoting invasion. *FEBS Letters*.

[35] Liu F. T., Dong Q., Gao H. (2017). The prognostic significance of UCA1 for predicting clinical outcome in patients with digestive system malignancies. *Oncotarget*.

[36] Sun X. D., Huan C., Qiu W. (2016). Clinical significance of UCA1 to predict metastasis and poor prognosis of digestive system malignancies: a meta-analysis. *Gastroenterology Research and Practice*.

[37] Li J. Y., Ma X., Zhang C. B. (2014). Overexpression of long non-coding RNA UCA1 predicts a poor prognosis in patients with esophageal squamous cell carcinoma. *International Journal of Clinical and Experimental Pathology*.

[38] Jiao C., Song Z., Chen J. (2016). lncRNA-UCA1 enhances cell proliferation through functioning as a ceRNA of Sox4 in esophageal cancer. *Oncology Reports*.

[39] Liu H. E., Shi H. H., Luo X. J. (2020). Upregulated long noncoding RNA UCA1 enhances Warburg effect via miR-203/HK2 Axis in esophagal cancer. *Journal of Oncology*.

[40] Wang X., Gao Z., Liao J. (2016). lncRNA UCA1 inhibits esophageal squamous-cell carcinoma growth by regulating the Wnt signaling pathway. *Journal of Toxicology and Environmental Health, Part A*.

[41] Zhu Z., Wang H., Pang Y., Hu H., Zhang H., Wang W. (2020). Exosomal long non-coding RNA UCA1 functions as growth inhibitor in esophageal cancer. *Aging*.

[42] Gao J., Cao R., Mu H. (2015). Long non-coding RNA UCA1 may be a novel diagnostic and predictive biomarker in plasma for early gastric cancer. *International Journal of Clinical and Experimental Pathology*.

[43] Zheng Q., Wu F., Dai W.-Y. (2015). Aberrant expression of UCA1 in gastric cancer and its clinical significance. *Clinical and Translational Oncology*.

[44] Gong P., Qiao F., Wu H. (2018). LncRNA UCA1 promotes tumor metastasis by inducing miR-203/ZEB2 axis in gastric cancer. *Cell Death & Disease*.

[45] Sun L., Liu L., Yang J., Li H., Zhang C. (2019). SATB1 3′-UTR and lncRNA-UCA1 competitively bind to miR-495-3p and together regulate the proliferation and invasion of gastric cancer. *Journal of Cellular Biochemistry*.

[46] Gu L., Lu L.-s., Zhou D.-l., Liu Z.-c. (2018). UCA1 promotes cell proliferation and invasion of gastric cancer by targeting CREB1 sponging to miR-590-3p. *Cancer Medicine*.

[47] Wang C. J., Zhu C. C., Xu J. (2019). The lncRNA UCA1 promotes proliferation, migration, immune escape and inhibits apoptosis in gastric cancer by sponging anti-tumor miRNAs. *Molecular Cancer*.

[48] Zuo Z.-K., Gong Y., Chen X.-H. (2017). TGF*β*1-Induced LncRNA UCA1 upregulation promotes gastric cancer invasion and migration. *DNA and Cell Biology*.

[49] Li C., Liang G., Yang S. (2017). Dysregulated lncRNA-UCA1 contributes to the progression of gastric cancer through regulation of the PI3K-Akt-mTOR signaling pathway. *Oncotarget*.

[50] Wang Z.-Q., Cai Q., Hu L. (2017). Long noncoding RNA UCA1 induced by SP1 promotes cell proliferation via recruiting EZH2 and activating AKT pathway in gastric cancer. *Cell Death & Disease*.

[51] Wang Z.-q., He C.-y., Hu L. (2017). Long noncoding RNA UCA1 promotes tumour metastasis by inducing GRK2 degradation in gastric cancer. *Cancer Letters*.

[52] Fang Q., Chen X., Zhi X. (2016). Long non-coding RNA (LncRNA) urothelial carcinoma associated 1 (UCA1) increases multi-drug resistance of gastric cancer via downregulating miR-27b. *Medical Science Monitor*.

[53] Cheng H., Sharen G., Wang Z., Zhou J. (2021). LncRNA UCA1 enhances cisplatin resistance by regulating CYP1B1-mediated apoptosis via miR-513a-3p in human gastric cancer. *Cancer Management and Research*.

[54] Yang Z., Shi X., Li C. (2018). Long non-coding RNA UCA1 upregulation promotes the migration of hypoxia-resistant gastric cancer cells through the miR-7-5p/EGFR axis. *Experimental Cell Research*.

[55] Hu J. J., Song W., Zhang S. D. (2016). HBx-upregulated lncRNA UCA1 promotes cell growth and tumorigenesis by recruiting EZH2 and repressing p27Kip1/CDK2 signaling. *Scientific Reports*.

[56] Hu M. L., Wang X. Y., Chen W. M. (2018). TGF-beta1 upregulates the expression of lncRNA UCA1 and its downstream HXK2 to promote the growth of hepatocellular carcinoma. *European Review for Medical and Pharmacological Sciences*.

[57] Zhao B., Lu Y., Cao X. (2019). MiRNA-124 inhibits the proliferation, migration and invasion of cancer cell in hepatocellular carcinoma by downregulating lncRNA-UCA1. *OncoTargets and Therapy*.

[58] Cui X., Zhao C., Yao X. (2018). SND1 acts as an anti-apoptotic factor via regulating the expression of lncRNA UCA1 in hepatocellular carcinoma. *RNA Biology*.

[59] Gao M., Li C., Xu M., Liu Y., Liu S. (2018). LncRNA UCA1 attenuates autophagy-dependent cell death through blocking autophagic flux under arsenic stress. *Toxicology Letters*.

[60] Xu Y., Yao Y., Leng K. (2017). Long non-coding RNA UCA1 indicates an unfavorable prognosis and promotes tumorigenesis via regulating AKT/GSK-3*β* signaling pathway in cholangiocarcinoma. *Oncotarget*.

[61] Kong L., Wu Q., Ye J., Li N., Yang H. (2019). Upregulated lncRNA-UCA1 contributes to metastasis of bile duct carcinoma through regulation of miR-122/CLIC1and activation of the ERK/MAPK signaling pathway. *Cell Cycle*.

[62] Cai Q., Jin L., Wang S. (2017). Long non-coding RNA UCA1 promotes gallbladder cancer progression by epigenetically repressing p21 and E-cadherin expression. *Oncotarget*.

[63] Chen P., Wan D., Zheng D., Zheng Q., Wu F., Zhi Q. (2016). Long non-coding RNA UCA1 promotes the tumorigenesis in pancreatic cancer. *Biomedicine & Pharmacotherapy*.

[64] Zhang X., Gao F., Zhou L., Wang H., Shi G., Tan X. (2017). UCA1 regulates the growth and metastasis of pancreatic cancer by sponging miR-135a. *Oncology Research Featuring Preclinical and Clinical Cancer Therapeutics*.

[65] Zhou Y., Chen Y., Ding W. (2018). LncRNA UCA1 impacts cell proliferation, invasion, and migration of pancreatic cancer through regulating miR-96/FOXO3. *IUBMB Life*.

[66] Zhang M., Zhao Y., Zhang Y. (2018). LncRNA UCA1 promotes migration and invasion in pancreatic cancer cells via the Hippo pathway. *Biochimica et Biophysica Acta (BBA)-Molecular Basis of Disease*.

[67] Liu Y., Feng W., Gu S. (2019). The UCA1/KRAS axis promotes human pancreatic ductal adenocarcinoma stem cell properties and tumor growth. *American Journal of Cancer Research*.

[68] Han Y., Yang Y.-n., Yuan H.-h. (2014). UCA1, a long non-coding RNA up-regulated in colorectal cancer influences cell proliferation, apoptosis and cell cycle distribution. *Pathology*.

[69] Bian Z., Jin L., Zhang J. (2016). LncRNA—UCA1 enhances cell proliferation and 5-fluorouracil resistance in colorectal cancer by inhibiting miR-204-5p. *Scientific Reports*.

[70] Cui M., Chen M., Shen Z., Wang R., Fang X., Song B. (2019). LncRNA-UCA1 modulates progression of colon cancer through regulating the miR-28-5p/HOXB3 axis. *Journal of Cellular Biochemistry*.

[71] Liu C., Ji L., Song X. (2019). Long non coding RNA UCA1 contributes to the autophagy and survival of colorectal cancer cellsviasponging miR-185-5p to up-regulate the WISP2/*β*-catenin pathway. *RSC Advances*.

[72] Cao C., Zhang J., Yang C., Xiang L., Liu W. (2020). Silencing of long noncoding RNA UCA1 inhibits colon cancer invasion, migration and epithelial-mesenchymal transition and tumour formation by upregulating miR-185-5p in vitro and in vivo. *Cell Biochemistry and Function*.

[73] Jahangiri B., Khalaj-Kondori M., Asadollahi E., Sadeghizadeh M. (2019). Cancer-associated fibroblasts enhance cell proliferation and metastasis of colorectal cancer SW480 cells by provoking long noncoding RNA UCA1. *Journal of Cell Communication and Signaling*.

[74] Yang X., Liu W., Xu X. (2018). Downregulation of long non-coding RNA UCA1 enhances the radiosensitivity and inhibits migration via suppression of epithelial-mesenchymal transition in colorectal cancer cells. *Oncology Reports*.

[75] Xian Z., Hu B., Wang T. (2020). lncRNA UCA1 contributes to 5-fluorouracil resistance of colorectal cancer cells through miR-23b-3p/znf281 Axis. *OncoTargets and Therapy*.

[76] Yang Y. N., Zhang R., Du J. W. (2018). Predictive role of UCA1-containing exosomes in cetuximab-resistant colorectal cancer. *Cancer Cell International*.

[77] Ramakrishnan P., Loh W. M., Gopinath S. C. B. (2020). Selective phytochemicals targeting pancreatic stellate cells as new anti-fibrotic agents for chronic pancreatitis and pancreatic cancer. *Acta Pharmaceutica Sinica B*.

[78] Wang F., Ying H.-Q., He B.-S. (2015). Upregulated lncRNA-UCA1 contributes to progression of hepatocellular carcinoma through inhibition of miR-216b and activation of FGFR1/ERK signaling pathway. *Oncotarget*.

[79] Zhao Z., Malhotra A., Seng W. Y. (2019). Curcumin modulates hepatocellular carcinoma by reducing UNC119 expression. *Journal Of Environmental Pathology, Toxicology And Oncology*.

[80] Wu Y. S., Looi C. Y., Subramaniam K. S., Masamune A., Chung I. (2016). Soluble factors from stellate cells induce pancreatic cancer cell proliferation via Nrf2-activated metabolic reprogramming and ROS detoxification. *Oncotarget*.

[81] Wu Y. S., Chung I., Wong W. F., Masamune A., Sim M. S., Looi C. Y. (2017). Paracrine IL-6 signaling mediates the effects of pancreatic stellate cells on epithelial-mesenchymal transition via Stat3/Nrf2 pathway in pancreatic cancer cells. *Biochimica et Biophysica Acta (BBA)-General Subjects*.

[82] Wei D. M., Jiang M. T., Lin P. (2019). Potential ceRNA networks involved in autophagy suppression of pancreatic cancer caused by chloroquine diphosphate: a study based on differentially-expressed circRNAs, lncRNAs, miRNAs and mRNAs. *International Journal of Oncology*.

[83] Sakamoto K. M., Frank D. A. (2009). CREB in the pathophysiology of cancer: implications for targeting transcription factors for cancer therapy: fig. 1. *Clinical Cancer Research*.

[84] Wu J., Li W., Ning J., Yu W., Rao T., Cheng F. (2019). Long noncoding RNA UCA1 targets miR-582-5p and contributes to the progression and drug resistance of bladder cancer cells through ATG7-mediated autophagy inhibition. *OncoTargets and Therapy*.

[85] Liu C., Jin J., Shi J., Wang L. (2019). Long noncoding RNA UCA1 as a novel biomarker of lymph node metastasis and prognosis in human cancer: a meta-analysis. *Bioscience Reports*.

[86] Sánchez Y., Huarte M. (2013). Long non-coding RNAs: challenges for diagnosis and therapies. *Nucleic Acid Therapeutics*.

[87] Lennox K. A., Behlke M. A. (2016). Mini-review: current strategies to knockdown long non-coding RNAs. *Journal of Rare Diseases Research & Treatment*.

[88] Bonam S. R., Wu Y. S., Tunki L. (2018). What has come out from phytomedicines and herbal edibles for the treatment of cancer?. *ChemMedChem*.

[89] Choudhari A. S., Mandave P. C., Deshpande M. (2020). Phytochemicals in cancer treatment: from preclinical studies to clinical practice. *Frontiers in Pharmacology*.

[90] Tuorkey M. J. (2015). Cancer therapy with phytochemicals: present and future perspectives. *Biomedical and Environmental Sciences*.

[91] Kamel M. M., Matboli M., Sallam M., Montasser I. F., Saad A. S., El-Tawdi A. H. F. (2016). Investigation of long noncoding RNAs expression profile as potential serum biomarkers in patients with hepatocellular carcinoma. *Translational Research*.

